# Detection of Chromosomal Breakpoints in Patients with Developmental Delay and Speech Disorders

**DOI:** 10.1371/journal.pone.0090852

**Published:** 2014-03-06

**Authors:** Kagistia H. Utami, Axel M. Hillmer, Irene Aksoy, Elaine G. Y. Chew, Audrey S. M. Teo, Zhenshui Zhang, Charlie W. H. Lee, Pauline J. Chen, Chan Chee Seng, Pramila N. Ariyaratne, Sigrid L. Rouam, Lim Seong Soo, Saira Yousoof, Ivan Prokudin, Gregory Peters, Felicity Collins, Meredith Wilson, Alyson Kakakios, Georges Haddad, Arnaud Menuet, Olivier Perche, Stacey Kiat Hong Tay, Ken W. K. Sung, Xiaoan Ruan, Yijun Ruan, Edison T. Liu, Sylvain Briault, Robyn V. Jamieson, Sonia Davila, Valere Cacheux

**Affiliations:** 1 Human Genetics, Genome Institute of Singapore, Singapore, Singapore; 2 Cancer Therapeutics and Stratified Oncology, Genome Institute of Singapore, Singapore, Singapore; 3 Stem Cells and Developmental Biology, Genome Institute of Singapore, Singapore, Singapore; 4 Computational and Mathematical Biology, Genome Institute of Singapore, Singapore, Singapore; 5 Scientific & Research Computing, Genome Institute of Singapore, Singapore, Singapore; 6 Eye and Developmental Genetics Research, The Children’s Hospital at Westmead, Children’s Medical Research Institute and Save Sight Institute, Sydney, New South Wales, Australia; 7 Disciplines of Paediatrics and Child Health and Genetic Medicine, Sydney Medical School, University of Sydney, Sydney, New South Wales, Australia; 8 Department of Cytogenetics, The Children’s Hospital at Westmead, Sydney, New South Wales, Australia; 9 Department of Clinical Genetics, The Children’s Hospital at Westmead, Sydney, New South Wales, Australia; 10 Department of Immunology, The Children’s Hospital at Westmead, Sydney, New South Wales, Australia; 11 Unité de Génétique, Centre Hospitalier, Blois, France; 12 Service de Genetique INEM UMR7355 CNRS-University, Centre Hospitalier Régional d’Orléans, Orléans, France; 13 Department of Paediatrics, Yong Loo Lin School of Medicine, National University of Singapore, Singapore, Singapore; 14 Genome Technology and Biology, Genome Institute of Singapore, Singapore, Singapore; University of Bonn, Institut of experimental hematology and transfusion medicine, Germany

## Abstract

Delineating candidate genes at the chromosomal breakpoint regions in the apparently balanced chromosome rearrangements (ABCR) has been shown to be more effective with the emergence of next-generation sequencing (NGS) technologies. We employed a large-insert (7–11 kb) paired-end tag sequencing technology (DNA-PET) to systematically analyze genome of four patients harbouring cytogenetically defined ABCR with neurodevelopmental symptoms, including developmental delay (DD) and speech disorders. We characterized structural variants (SVs) specific to each individual, including those matching the chromosomal breakpoints. Refinement of these regions by Sanger sequencing resulted in the identification of five disrupted genes in three individuals: guanine nucleotide binding protein, q polypeptide *(GNAQ),* RNA-binding protein, fox-1 homolog *(RBFOX3),* unc-5 homolog D (*C.elegans) (UNC5D*), transmembrane protein 47 (*TMEM47*), and X-linked inhibitor of apoptosis (*XIAP*). Among them, *XIAP* is the causative gene for the immunodeficiency phenotype seen in the patient. The remaining genes displayed specific expression in the fetal brain and have known biologically relevant functions in brain development, suggesting putative candidate genes for neurodevelopmental phenotypes. This study demonstrates the application of NGS technologies in mapping individual gene disruptions in ABCR as a resource for deciphering candidate genes in human neurodevelopmental disorders (NDDs).

## Introduction

Apparently balanced chromosomal rearrangements (ABCR) occur sporadically in the population or segregate within families, with a frequency of 1 in every 2000 live births. ABCR have been largely associated with infertility, ovarian failure and intellectual disability (ID) when detected during pre- and post-natal investigation or genetic counselling. [1], [2], [3], [4], [5], [6] The risk of developing congenital anomalies or NDDs has been estimated to be 6.1% for *de novo* ABCR. [2] Although ABCR is also found in phenotypically normal individuals, there is an increased incidence of ABCR in NDDs patients, which may lead to novel candidate disease gene identification through breakpoint cloning methods. Such method has been successfully applied in characterizing disease genes including *DMD* in Dystrophin for Duchenne Muscular Dystrophy [7], *DISC1* in Schizophrenia [8], [9] and *ATP7A* in Menkes disease. [10], [11] Karyotyping, Array-CGH (aCGH) and SNP arrays are currently first-tier diagnostic tools to investigate ABCR in pre- and post-natal settings. [6], [12], [13] Balanced chromosomal aberrations can only be detected through karyotype observation, although at a low resolution of approximately 5–10 Million base-pairs (Mb), [14] and subsequent Fluorescence *in situ* hybridization (FISH) mapping across the breakpoints for more detailed delineation is restricted to the investigated region. Microarrays technologies are able to identify copy number changes at considerable resolution but fail to identify copy number neutral rearrangements. [15], [16] Recent advances in NGS technologies render it possible to systematically identify genomic rearrangements including copy number neutral events at a base-pair resolution, thus facilitating candidate disease gene identification in the chromosomal breakpoint regions.

We have established a NGS pipeline, referred to as a DNA-PET sequencing, [17], [18], [19] in which we sequence the short ends (50 bp) of 5′ and 3′ tags of large-insert sizes between 7 to 11 kb genomic DNA fragments, followed by ligating the two paired-ends to form PET constructs and subjected to sequencing in a massive and highly parallel manner, (Figure S1, see Supporting Information S1) [17], [20]. Hillmer and colleagues [17], [18], [19] have demonstrated that large-insert fragment sizes provided higher physical coverage with minimum sequencing efforts, and have the advantage over short-insert sizes in terms of large genomic SVs detection and covering complicated DNA sequence features such as repetitive regions. We implemented this technique to analyze the genome of four individuals with NDDs symptoms including Developmental Delay (DD), Speech Delay (SD), Language Delay (LD) and autistic disorder. These patients harbour cytogenetically defined ABCR (2 familial translocation, 1 familial inversion and 1 *de novo* inversion), and prior aCGH analysis did not reveal chromosomal imbalances. Our study shows that NGS technology enables rapid identification of individual gene disruptions and potential candidate genes in ABCR. We also demonstrated the correlation of disrupted gene *XIAP* in an inversion breakpoint to be causative for the patient’s immunodeficiency phenotype.

## Results

### Structural Variants Detection by DNA-PET Sequencing in Four Patients with Developmental Delay and Speech Disorders

By using patients’ genomic DNA as starting material, libraries were generated and sequenced using a SOLiD platform, and we obtained an average of 35 million non-redundant paired-end reads (Table S1, see Supporting Information S1). The median physical coverage using our technique was 98x, with an average of 94x (Table S1, see Supporting Information S1). Majority of the Paired End Tags (PETs) were mapped to the reference genome NCBI Build 36 and referred to as concordant PETs (cPETS), which provided the copy number information based on sequencing read-depth. The remaining clustered PETs were referred to as discordantly mapped PETs (dPETs), which allowed the identification of structural variants (SVs). After filtering (see Methods section), approximately 96% of the SV calls generated for each library were shared with normal individuals published in the Database of Genomic Variants [21], 1000 Genome Project SVs Pilot release set [22], [23] or previous paired-end sequencing studies of normal individuals [24], [25], [26].

Using our analysis pipeline for patient-specific SVs discovery, the extraction of normal SVs reduced this number to 7–19 events, with a mean of 14 SVs per patient (Tables S2–S6, see Supporting Information S1). We observed deletions as being the most frequent SVs, comprising 58% of the total patients-specific SVs (32 deletions out of a total of 55 SVs), while tandem duplication comprises 20%, and the remaining SVs (translocation, inversion and insertion) comprise 21% of total SVs (Table 1). We performed PCR analysis on 36 randomly chosen SVs with 2 sets of primers spanning predicted breakpoint junctions. Of these, 27 SVs were validated and produced a single, clear PCR band at expected size range, suggesting ∼75% validation rate. In parallel to DNA-PET, we compared the copy number detection overlap to aCGH and observed an average of 93% overlap between both experiments (Table S7, see Supporting Information S1).

**Table 1:** List of SVs obtained after filtration of four patients.

						Patient-specific SVs after filtration							
Family	Patient	Karyotype	dPETclusters	SVs after datacuration	SVs overlap withnormal	Total	Del	BalanceTransloc.	IsolatedTransloc.	UnpairedInversion	PairedInversion	TandemDuplication	Insertion
**1**	CD5	46, XY,t(9;17)(q12;q24)	505	284	270 (95.07%)	14	12	2	0	0	0	0	0
**2**	CD10	46,XY, t(6;8)(q16.2;p11.2)	927	616	601 (97.50%)	15	9	2	1	0	0	1	2
**3**	CD8	46,XY,inv(X)(p22q26)	724	387	368 (95.09%)	19	9	0	0	1	2	7	0
**4**	CD9	46,XY,inv(5)(q22q35.1)	930	581	574 (98.79%)	7	2	0	0	0	2	3	0
**Total**			3,086	1,868	1,813	55	32 (58.1%)	4 (7.2%)	1 (1.8%)	1 (1.8%)	4 (7.2%)	11 (20%)	2 (3.6%)

a Discordantly mapped PETs (dPETs) which connect the same two genomic regions are clustered together.

b dPET clusters which has passed quality filters to remove sequencing artefacts provided the list of predicted SVs.

c Approximately 95% of SVs overlapped with SVs found in normal population (see Methods).

For each patient, we performed a case by case study to identify the potential genes which were disrupted by the cytogenetically visible rearrangements with the assumption that these were the most likely causative events. We validated DNA-PET breakpoints matching the cytogenetic rearrangements by Sanger sequencing (Table 2), FISH and evaluated gene expression by quantitative real-time PCR (RT-PCR). Apart from the specific chromosomal rearrangements, we checked other SVs obtained for each patient. Most of the SVs that coincide with coding regions have been reported in the Database of Genomic Variants (DGV), indicating that these SVs are likely benign (Tables S2–6, see Supporting Information S1). We checked for the presence of regulatory elements, such as transcription factor binding sites and epigenetic marks, using RegulomeDB and the ENCODE dataset, on SVs present in intra/intergenic regions and found no evidence of known regulatory elements in any of them.

**Table 2:** Validated breakpoints and disrupted candidate genes from DNA-PET sequencing in four patients.

Patient	Cytogenetic Analysis	Chr.	DNA-PET breakpoint predicted coordinate (SOLiD)	Validated breakpoint coordinate (Sanger)	Gene
CD5	t(9;17)	9	79,571,716–79,573,787	79,572,001–79,572,005	*GNAQ*
		17	74,764,804–74,768,401	74,764,927–74,767,964	*RBFOX3*
CD10	t(6;8)	6	98,318,059–98,318,840	98,318,526–98,318,538	
		8	35,527,808–35,528,282	35,527,964–35,527,976	*UNC5D*
CD8	inv(X)	X	119,984,597–119,984,844	119,984,613–119,984,615	
		X	122,839,027–122,845,538	122,839,398–122,844,862	*XIAP*
CD9	inv(5)	5	111,962,591–111,963,149	111,962,767	
		5	165,575,819–165,576,628	165,576,568–165,576,574	

From these extensive analyses, we identified five disrupted genes in three patients: *GNAQ, RBFOX3, UNC5D, XIAP* and *TMEM47* (Table 2). Among them, *XIAP* has already been associated with X-linked lymphoproliferative disorder 2 (XLP-2: MIM [300635]). To evaluate the potential implication of the disrupted genes, we assessed the occurrence of pre-existing Copy Number Variations (CNVs) in DD cases from published studies and the DECIPHER Consortium [27], [28], [29] (Table 3). Enrichment of CNV counts in the cases versus controls was observed in *RBFOX3* and *UNC5D,* suggesting that altered gene dosage in these genes might have a role in NDDs.

**Table 3:** Screening of CNVs in cases/controls from published and public datasets.

		Cases			Control		
Patient	Disrupted genes	Cooper et al [28]	DECIPHER Consortium	Total	Cooper et al [28]	1000 Genome	Total
**CD5**	*GNAQ*	3	7	10	14	0	14
**CD5**	*RBFOX3*	68	11	79	1	4	5
**CD10**	*UNC5D*	2	15	17	0	0	0
**CD8**	*TMEM47*	0	10	10	0	4	4

The total number of cases in Cooper et al [28] is 15,767 cases, and for DECIPHER is ∼17,000 cases [29]. The total number of controls in Cooper et al. is 8329 controls and for 1000 Genome SV Release set is 185 controls [22].

### Breakpoint Characterization through Detailed SVs Analysis

#### Case 1 (Patient CD5)

**Figure 1:**
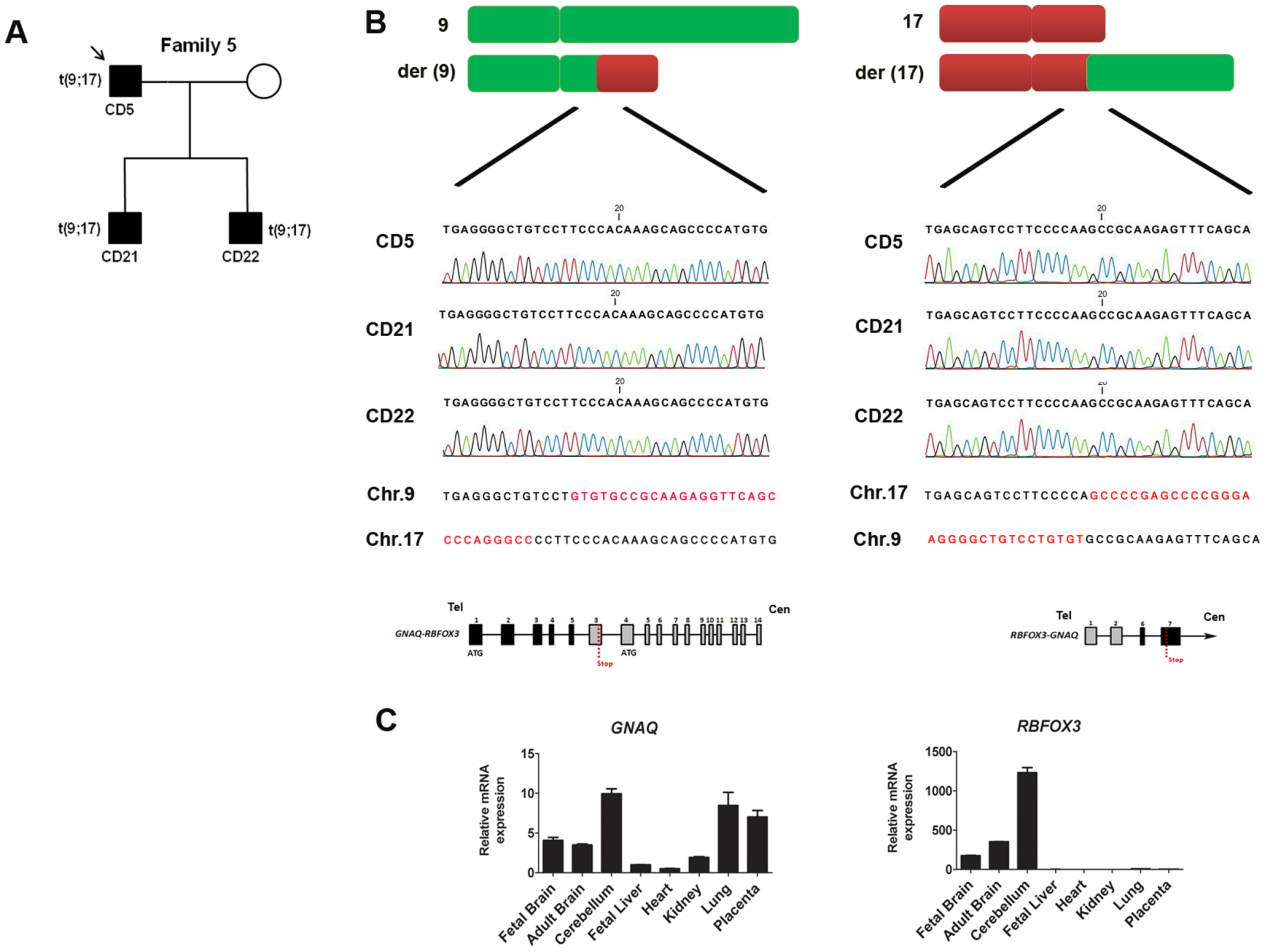
Patient CD5 with translocation t(9;17). A) The pedigree of patient CD5 is indicated. The translocation is transmitted to his two sons (CD21 and CD22). B) Translocation between chromosome 9 and 17 were validated by Sanger sequencing in three translocation carriers. The reference sequence is indicated, showing the fusion of two genes at the genomic level: the first five exons of *GNAQ* fused to exon 3–14 of *RBFOX3* and the first two exons of *RBFOX3* fused to exon 6–7 of *GNAQ.* C) mRNA expression of *GNAQ* and *RBFOX3* showed high expression in fetal brain, adult brain and cerebellum in human tissue panel.

In this family, the translocation segregates with a variable degree of phenotypic manifestation. Patient CD5 had language delay (LD) and DD during his childhood, and his two sons (CD21 and CD22) displayed absence of speech, DD and autistic disorder (Figure 1A). The translocation t(9;17) was represented by two abnormally oriented clusters corresponding to the balanced translocation between chromosome 9 and 17. Sanger sequencing refined the breakpoint coordinates in the translocation carriers, and revealed identical breakpoint patterns on chromosome 9 at position chr9∶79,572,001–79,572,005 and on chromosome 17 at position chr17∶74,764,927–74,767,964. There is a loss of 4 bp and 3,036 bp on chromosome 9 and 17, respectively, with a microhomology of 3 bp between paired breakpoints (Figure 1B). The translocation disrupted *GNAQ* at intron 5 on chromosome 9 and *RBFOX3* at intron 2 on chromosome 17. Both disrupted genes shared the same orientation, breakpoints lie within introns, and the resulting fusion is predicted to be in frame. However, RT-PCR analysis on the patient’s lymphoblastoid cell line did not reveal any fusion transcript expression as neither gene is expressed in these cells, reflecting the importance of relevant cell type for validation of brain-specific genes. We checked the mRNA expression of both genes in human tissue panel and found that both genes are highly expressed in the fetal brain and the cerebellum (Figure 1C). *GNAQ* encodes a member of the Gαq heterotrimeric protein family, and null *Gnaq* mice exhibit cerebellar ataxia, and motor coordination deficit [30]. *RBFOX3* is a neuronal specific splicing factor, which is exclusively expressed in the neuronal nuclei. There are 12 other SVs found in this patient, and nine of them were validated by PCR, in which 5 SVs were shared with his two affected sons (SV1, 2, 4, 6 and 13). These five SVs overlapped with known CNV regions (SV1, 2, 4) reported in healthy individuals or were in intergenic regions (SV6, 13) and were therefore excluded for further analysis (Table S2, see Supporting Information S1). Overall, the translocation segregates with LD and DD in this family with variable penetrance, hinting a potential functional impact.

#### Case 2 (Patient CD10)

**Figure 2:**
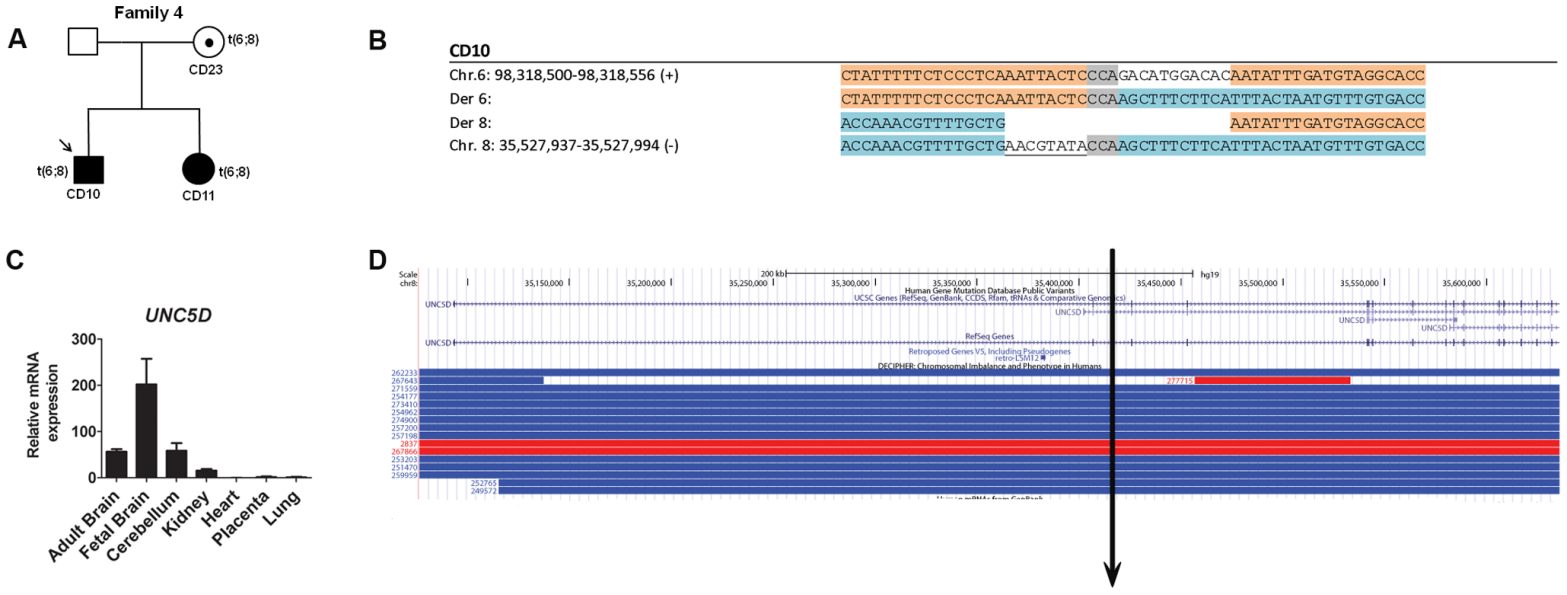
Patient CD10 with translocation t(6;8). A) The pedigree of patient CD10 is indicated. The familial translocation is inherited from asymptomatic carrier mother and shared with his affected sister (CD11). B) Sanger sequencing analysis refined the chromosomal breakpoint regions and revealed a loss of 11 bp on chromosome 6 and 8 bp on chromosome 8, with a microhomology of 3 bp between the paired breakpoints. C) *UNC5D* mRNA expression in human tissue panel showed high expression in the fetal brain, adult brain and cerebellum compared to other tissues. D) The translocation breakpoint is located at intron 1 of *UNC5D* indicated by the black arrow, encompasses 15 CNVs cases described in the DECIPHER.

In the second patient (CD10), the causative effect of the t(6;8) balanced translocation is unclear, due to variable expression of phenotype in the translocation carriers; his mother is asymptomatic and his younger sibling displayed schizencephaly and DD (CD11) (Figure 2A). DNA-PET identified abnormal paired reads matching the balanced translocation, and Sanger sequencing refined the breakpoint coordinates on chromosome 6 at position chr6∶98,318,526–98,318,538 and chromosome 8 at position chr8∶35,527,969–35,527,976. The translocation disrupted *UNC5D* at intron 5 on chromosome 8, while no coding genes were disrupted on chromosome 6 (Figure 2B). *UNC5D* encodes a member of human dependence receptor UNC5 family that is specifically expressed in the layer 4 of the developing neocortex in rats, which makes it a biologically plausible candidate [31], [32]. Since the translocation does not fully segregate with the disease, all coding exons of *UNC5D* were sequenced in each translocation carriers, and we did not find additional point mutations in the other allele. *UNC5D* mRNA is exclusively expressed in the adult brain, fetal brain, and cerebellum (Figure 2C), thus expression changes could not be investigated in the patient’s lymphoblastoid cell line. Notably, we observed enrichment of CNV counts in 15 cases described by DECIPHER (Figure 2D). Analysis of other SVs showed 5 deletions in intergenic regions (SV1, 2, 11, 12, and 14) and 8 within genes (SV3, 4, 5, 6, 9, 10, 13 and 15), in which all of them overlapped with known CNVs regions (Table S3, see Supporting Information S1).

#### Case 3 (Patient CD8)

**Figure 3:**
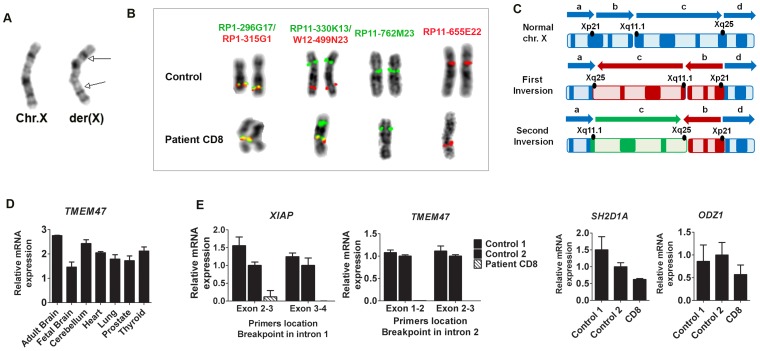
Patient CD8 with a complex chromosomal inversion. A) Karyogram of normal chromosome X compared to der(X) in patient CD8. B) FISH validation of 10 SVs shown in Table S5 (see Supporting Information S1) with the respective FISH probes: Hybridization of RP1-296G17-Biot (SV15) and RP1-315-Dig (SV16) were localized on the centromere of the patient’s metaphase. Probes for SV17 and SV21 on Xp21 (RP11-330K13-Biot) and Xq25 (W12-499N23-Dig), respectively resulted in a split signal between Xp21 and the centromeric region in the patient’s chromosome. Further FISH analysis was performed by using probe RP11-762M23-Biot on Xq11.1 (SV22) that was found to localize on the upper chromosomal arm. Probe RP11-655E22 on Xp11.2 was localized on the lower arm of derivative chromosome X. C) Reconstructed derivative chromosome X for patient CD8. Normal human chromosome X according to ISCN 2009 with the arrow orientation from *a* to *d* and the proposed mechanism of sequential double inversion in patient CD8. Based on our FISH analysis, an inversion occurred first between Xp21 and Xq25, changing the orientation of p and q arm with a shift of the centromere position towards the lower q-arm shown by inverted red arrow *b* and *c*. This was followed by the second inversion that occurred between Xq11.1 and Xq25, altering the orientation of the q-arm (inverted green arrow *c*). D) Expression of *TMEM47* in human tissue panel assessed by qRT-PCR. E) Expression analysis of four disrupted genes in patient CD8 assessed by qRT-PCR.

The third patient (CD8), carried a maternal-derived pericentric inversion at chromosome X, between Xp22 and Xq26 (Figure 3A). His monozygotic twin brother harbors the same inversion. They both show signs of immunodeficiency combined with mild intellectual disability (ID) and LD. SVs analysis of this patient revealed a 1.2 Mb inversion located at chromosome Xq24–25 between paired breakpoints (Table S4, see Supporting Information S1), which did not correspond to the large inversion seen in the karyotype, inv(X)(p11.4;q24). Sanger sequencing allowed us to delineate the precise breakpoint on chromosome X at position chrX:119,984,613–119,984,615 and at position chrX:122,839,398–122,844,862 with a loss of 1 bp and 5,463 bp on each breakpoint junction, respectively. Due to the discrepancies between karyogram and NGS data, we traced other SVs in chromosome X by including lower confidence SVs (cluster size ≥4), and revealed a total of 10 SVs that clustered into multiple breakpoint hotspots on the p arm (SV14, 17, 20, 21), the centromeric region (SV22) and the q arm (SV13, 15, 16, 18, 19), suggesting a complex chromosomal rearrangement mechanism (Table S5, see Supporting Information S1). We verified the breakpoints in the inversion carriers (the mother and the twin boys) by PCR and FISH using probes RP1-315G1 (Xq25), RP1-296G17 (Xq24), RP11-330K13 (Xp21), W12-499N23 (Xq25) BAC and Fosmid clones (Figure 3B). These data suggested two large sequential breaks in each arm of chromosome X (Xp21 and Xq24–25) that resulted in tandem duplications, deletion and isolated breakpoints (Figure 3C). This double-inversion mechanism rearranged the landscape of chromosome X architecture, and appeared as a large inversion on G-banding karyotype. Combining DNA-PET sequencing and extensive FISH validations allowed us to characterize the complex chromosomal rearrangements in chromosome X, as a result of a for a sequential double inversion mechanism.

This complex rearrangement disrupted two genes: *XIAP* and *TMEM47*. The latter encodes a member of PMP22/EMP/Claudin protein family is ubiquitously expressed in human tissues including adult and fetal brain (Figure 3D). Two other genes were affected by tandem duplications as part of the complex rearrangement (SV18, 19): *SH2D1A*, encodes an SH2 domain containing 1A protein (SAP), which has been associated with X-linked lymphoproliferative syndrome type 1 (XLP-1 [MIM: 308240]). This gene was initially suspected as the primary cause of immunodeficiency phenotype seen in the patient, although it was rejected due to normal SAP expression in patient’s blood lysates (see Patients and Methods). *ODZ1* is the second gene affected by this rearrangement. It encodes an Odd Oz/Ten-M homolog 1 of Drosophila pair-rule gene involved in post-segmentation processes, including embryonic development of the central nervous system (CNS), eyes, and limbs in Drosophila [33], [34].

RT-PCR of the four genes revealed a total absence of expression for *XIAP1* and *TMEM47* and a moderate to normal expression for *SH2D1A* and *ODZ1* in patient’s fibroblast cell line compared to sex-matched controls, supporting the absence of functional *XIAP1* and *TMEM47* genes in the male patient (Figure 3E). Considering the normal expression of SAP protein and the absence of functional gene expression of *XIAP*, these data clearly suggest that XLP-2 underlies the immunodeficiency symptoms of the affected twin boys.

#### Case 4 (Patient CD9)

In patient 4 (CD9), sequencing analysis identified two paired-inversion clusters corresponding to the large inversion on chromosome 5. This inversion spans 53 Mb on chromosome 5q22.2 (chr5∶111,966,591-111,963,149) and 5q34 (chr5∶165,575,819–165,576,628), with 1 bp deletion, and microhomologies of 3 bp between paired breakpoints. No gene was disrupted at the breakpoint junctions (Table S6, see Supporting Information S1), and no evidence of regulatory elements sitting at both breakpoint coordinates was found based on RegulomeDB and ENCODE databases. Additionally, there are 5 other patient-specific SVs; two were located in the intergenic regions (SV5 and 6); two others were tandem duplications overlapped with known CNVs (SV4 and 7); and one intronic deletion of 4 kb in *RNF19B* gene, which has not been listed in DGV (SV1) (Table S6, see Supporting Information S1). Based on this analysis, it seems unlikely that the large inversion on chromosome 5 causes the clinical phenotype. Other patient specific SVs or mutations that cannot be identified by DNA-PET such as point mutations or exposures of environmental factors might be the underlying cause of the phenotypic features seen in this patient.

## Discussion

In the past decade, we have seen substantial progresses for identification of novel candidate genes in NDDs with the recent development in technologies. Two studies of large cohorts of patients with developmental delay described the enrichment of large CNVs in 15% of the cases [28], and highlighted the presence of additional large CNVs that co-exist with primary microdeletion/duplication syndrome in 10% of the cases as an additive contributing factor to more severe phenotype [35]. These CNVs have been useful to provide a better classification of microdeletion/duplication syndromes; however these regions often encompass multiple genes and thus make it challenging to identify plausible candidates. Recent exome sequencing study in individuals with ID identified potentially causative *de novo* single nucleotide variants (SNVs) with a diagnostic yield of 16%, comparable to the CNV burden obtained in copy number studies [36]. A more conventional approach in candidate gene identification involves delineating candidate genes in the chromosomal breakpoints of the ABCR [9], [37], [38], [39]. In contrast to the downstream effects of SNVs or CNVs, genes that are disrupted by translocations or inversion are presumably more severely affected, resulting generally in protein truncation or heterozygous inactivation of the affected allele.

Recent studies have described the feasibility of using NGS technologies to map the ABCR breakpoints in patients with neurodevelopmental abnormalities [37], [38], [40], [41], [42], [43], [44]. These technologies include (i) a shotgun sequencing approach by using the flow-sorted derivative chromosomes [40], (ii) custom jumping libraries coupled to targeted breakpoint capture [38], which are limited to the chromosomal breakpoint regions, (iii) standard paired-end sequencing or large-insert jumping libraries of 3–4 kb [37], [38], [41], and (iv) mate-pair sequencing of 2–3 kb insert sizes [42], [43].

Our work described the use of a genome paired end tag (DNA-PET) sequencing with larger insert sizes between 7–11 kb [17], [18], [19], [45], [46]. The use of an approximately 8–15 kb insert sizes has been shown to be more advantageous in terms of SVs detection in more complicated DNA sequence features, such as repetitive regions or large genomic rearrangements, and also provides higher physical coverage with minimum sequencing efforts compared to smaller insert sizes [19], [47]. We implemented this technique to map the breakpoints of four patients with DD harbouring cytogenetically defined ABCR, and identified specific breakpoints for all of them. Sanger sequencing was required to refine the breakpoints at the base pair level. The observed cryptic breakpoint anomalies were deletions ranging from 3 bp to 5,462 bp and/or microhomologies of 2–4 bp suggesting a microhomology-mediated end joining (MMEJ) [48] or non-homologous end joining mechanism (NHEJ) [49]. Both are major pathways for double strand break repair that often occurs in non-homologous regions of non-recurrent chromosomal breakpoints emphasizing the unique characteristics of these rearrangements.

In this study, five genes were identified within the ABCR-breakpoint regions in three patients (*GNAQ, RBFOX3, UNC5D, XIAP,* and *TMEM47*), and we observed various complications in attempting to correlate these genes to the expressed phenotypes. For one case (Patient CD8), the complex inversion pattern seen in chromosome X resembles chromothripsis, phenomenon commonly found in cancer and recently, in congenital diseases, which resulted from localized shattering of one or few chromosomes and assembly of chromosomal pieces by non-homologous end-joining (NHEJ), and thus appeared as complex chromosome rearrangements, involving three or more breakpoints [41], [42], [43], [50], [51]. This complex events highlight the advantage but also the limitation of the DNA-PET technology as FISH experiments were necessary to better understand the high order structure of the rearrangement. The inversion breakpoint disrupted *XIAP* gene, which is the primary cause of a rare form of XLP2 [52], [53] (XLP2 [MIM: 300635]) and likely to be causative for the immunodeficiency phenotype of the patient. More interestingly, these patients also presented mild ID and LD, features that are occasionally not associated with XLP syndromes, suggesting possibilities of additional contributing candidate genes within the complex rearrangements. *TMEM47* is among the potentially interesting candidate, with total absence of transcript expression in patient’s cell line and expression in fetal to adult brain. We proposed the need to perform mutational screening of *TMEM47* in X-linked ID patients for future studies.

For one patient (Patient CD9), there were no disrupted genes or regulatory elements at the breakpoint regions, neither potential other interesting SVs, showing the limitation for candidate gene detection merely through ABCR breakpoint cloning methods. Alternatively, exome sequencing of parent-offspring trio can be considered as a method of choice to further investigate possible causal variants especially in cases where there is a lack of association between ABCR and disease.

For the two remaining familial translocations, we identified fusion gene at the genomic level, although unverifiable at the transcript level due to lack of expression in available cell lines between *GNAQ* and *RBFOX3* genes in a t(9;17) translocation in three affected members of a family (Patient CD5) with different level of neurological symptoms (mild to severe) and *UNC5D* disruption in a family harbouring a t(6;8) translocation carried by two affected siblings (Patient CD10) and his mother. The translocation carriers in latter family displayed a broad range of clinical presentations; an asymptomatic mother, her first child with mild DD, and her second child presenting schizencephaly, polymicrogyria and LD, suggesting additional etiological factor underlying these features apart from the t(6;8) translocation. Besides the large phenotypic variability between translocation carriers seen in both families, we observed an enrichment of CNV counts for *UNC5D* and *RBFOX3* in NDDs-associated cases [28]. Therefore, we cannot totally exclude the possibility of high level of variability effects in these genes. Independent validation screening in isolated neurologically-affected patients, rather than collective multi-symptoms cohorts, would be significantly useful to provide significant association with specific neurodevelopmental symptoms in these patients.

Despite the potential functions of four identified genes (*GNAQ, RBFOX3, UNC5D* and *TMEM47)* in brain development, further functional validations are required to establish a correlation between these disrupted genes and patients’ clinical phenotype. Furthermore, reporting such ABCR-disrupted genes is crucial to determine the clinical relevance of newly identified CNVs or SNVs encompassing these genes.

In this study, we showed the potential of a cost-effective NGS technology to rapidly pinpoint disrupted genes within ABCR breakpoint regions. High overlap (93%) between both NGS-and array-based technologies shown in this study emphasizes the importance of employing a single technique that can provide high coverage of genome information with consistent validation rate as a routine clinical diagnosis tool. The stringency of our filtering pipeline can be optimized and adjusted depending on the sequencing platforms, and this would revolutionize the characterization of individual genomes of patients without prior karyotypic observation. For the implementation in the clinical settings, unaffected parents or siblings should be included for genome investigation to reduce the number of familial, non-pathogenic SVs. Whilst the technology continues to improve, validation with longer read depth by Sanger sequencing is still necessary for better SVs annotation. Our study complements the existing application of NGS technology in unexplained NDDs patients for better characterization of chromosomal rearrangements and discovery of potential candidate genes.

## Patients and Methods

### Patients

All samples and information were collected after written informed consent from patient’s parents was obtained and in accordance with local institutional review board approved protocols from National University of Singapore in Singapore, Children’s Hospital Westmead in Sydney, Australia and Centre Hospitalier Regional in Orleans, France. DNA samples were obtained from peripheral blood lymphocytes and cultured skin fibroblasts obtained from patients seen at the participating institutes.

#### Patient CD5

This is a familial balanced translocation presenting variable degree of DD and autistic features. The first son displayed an autistic behavior and global DD at three years of age with an absence of speech, feeding and sleeping difficulties, habit disorders, and stereotypic movements. At 4 years, there is an improvement in communication and speech seen in the first son. Chromosome analysis revealed a translocation t(9;17) (see Table 1), which is shared with his father and sibling. During genetic counselling, the father reported that he suffered from LD and DD during childhood that was not explored at that time, and this has resolved by adolescence. His second son was found to have DD and autistic features at the age of two years. DNA tested for the translocation was obtained from the father who was referred to as patient CD5. Chromosome analysis in the phenotypically normal mother revealed a normal karyotype.

#### Patient CD10

The patient was born at term by lower segment caesarean section, due to breech presentation. Delay in developmental milestones was noted at 18 months of age affecting both walking and speech. At 9 years old, comprehension and behavioural difficulties were noted at school. Karyotype analysis revealed a balanced translocation t(6;8) (see Table 1). Metabolic screening and FRAXA testing were normal. Karyotypic analysis in his parents revealed that his mother carried the same balanced translocation. She had no intellectual difficulties, but was reported to have had a ‘hole in the heart’ in childhood, which closed spontaneously. His younger sister had the same translocation detected by amniocentesis during pregnancy. She was noted to have plagiocephaly soon after birth. She had feeding difficulties in early infancy, which gradually resolved. At 2 years old, she had LD with low-average fine motor and gross motor skills. A right intermittent exotropia was noted. An MRI head scan showed a closed lip schizencephaly involving the right frontal lobe with polymicrogyria in the right Sylvian fissure. MRI brain scans in her brother and mother were normal.

#### Patient CD8

The patient was one of monozygotic twins. At the age of three years, he had an acute EBV infection with prolonged hepatosplenomegaly and abnormality of liver function tests. Immunological investigations showed reduced IgM, decreased CD4 T-helper cells and decreased natural killer (NK) cell function. Patient was suspected for XLP-1, but western blot analysis on patient’s whole blood cell lysates showed normal expression of SAP. At the age of five years, mild ID was diagnosed, as well as a specific language disorder affecting his receptive and expressive skills. Karyotype analysis revealed a complex inversion inv(X) (see Table 1), derived from his phenotypically normal mother. His identical twin showed similar clinical features and carrying the same karyotype. The maternal uncle was reported to have recurrent infections and a maternal great uncle died at eight months of age, apparently due to liver abnormalities.

#### Patient CD9

The patient was born by normal delivery after an uneventful pregnancy. Mild delay in early motor and speech developmental milestones was reported. At age twelve, he was found to have average intellectual ability, an expressive language disorder, and specific learning difficulties in maths, spellings and reading; as well as attention deficit disorder. Chromosome analysis revealed a paracentric inversion of chromosome 5 (see Table 1). Both parents have normal karyotypes and do not show any phenotypic abnormality.

### Cytogenetic and FISH Analysis

Karyotypes were determined from G-banding analysis using standard protocol according to the ISCN nomenclature. FISH analysis was carried out using protocols as described elsewhere. [54], [55] Metaphase chromosomes were prepared from Epstein-Barr virus-transformed lymphoblastoid cell lines (EBV-LCL) or cultured skin fibroblast cells obtained from patients, parents or siblings carriers by standard techniques. BAC and fosmid probes were obtained from BACPAC Resources (Oakland, CA). Probes were labelled by nick-translation kit (Enzo) with biotin-16-dUTP or digoxigenin-11-dUTP (Roche). The probes were blocked with 1 µg/µl Cot-1 DNA (Life Technologies), and resuspended at a concentration of 5 ng/µl in hybridization buffer (2xSSC, 10% Dextran Sulfate, 1x PBS, 50% Formamide). Fluorescent signals were visualized by avidin-conjugated fluorescein isothiocyanate (FITC) (Vector Laboratories, CA) or anti-Digoxigenin-Rhodamine (Roche). Chromosomes were counter-stained with DAPI and the signal was analysed using a Nikon Epifluorescence Microscope equipped with ISIS Metasystems for imaging analysis.

### Genomic DNA Preparation

Genomic DNA from lymphoblastoid cell lines, fibroblast, or blood from the patient was extracted by Qiagen Blood and Cell Culture DNA Kits (Qiagen), according to manufacturer’s instruction. lymphoblastoid and fibroblast cell lines were maintained to a minimum number of passages prior to DNA extraction. Quality and quantity of the extracted DNA were measured using NanoDrop 1000 Spectrophotometer and agarose gel electrophoresis.

### aCGH

aCGH was performed in all patient samples using the SurePrint G3 Human 2 x 400 k aCGH Microarray (Agilent Technologies Inc. Santa Clara) according to the manufacturer’s instruction. The microarray slides were scanned on an Agilent Microarray Scanner. Data were processed by Genomic Workbench software, standard edition 5.0.14 (Agilent). We used the Aberration Detection Method (ADM-2) algorithm to identify DNA copy number variations (CNV). The ADM-2 algorithm identifies all aberrant intervals in a given sample with consistently high or low log ratio based on the statistical score. We applied a filtering option of minimum of three probes in region and centralization threshold of 6. We used NCBI Build 36 as a reference genome. For smaller CNVs that were identified by DNA-PET, we compared with the aCGH raw data and interpreted the copy number change by looking at the individual probe ratio, using a cutoff of 0.1 (<0.1 indicated deletion, >0.1 indicated duplication).

### DNA-PET Library Construction

We constructed the DNA-PET libraries for four patients according to Method described in Hillmer et al. [17]. Briefly, genomic DNA was hydrosheared to 7–11 kb DNA fragments. Long Mate Paired (LMP) cap adaptors were ligated to the hydrosheared and end-repaired DNA fragments. The cap adaptor-ligated DNA fragments were separated by agarose gel electrophoresis, recovered using the QIAEXII Gel Extraction Kit (QIAGEN) and circularized with a biotinylated adaptor that connects the cap adaptors at both ends of the DNA fragments. Missing 5′ phosphate groups of cap adaptors created a nick on each strand after circularization of the DNA. Both nicks were translated outwards by >50 bp into the circularized genomic DNA fragment by DNA polymerase I (NEB). The nick-translated constructs were then digested with T7 exonuclease and S1 nuclease (NEB), to release paired-end tag (PETs) library constructs. These constructs were ligated with SOLiD sequencing adaptors P1 and P2 (Life Technologies), and amplified using 2x HF Phusion Master Mix (Finnzymes OY) for sequencing. High throughput sequencing of the 50 bp libraries was performed on SOLiD sequencers (v3plus and v4, respectively) according to the manufacturer’s recommendation (Life Technologies). Sequence tags were mapped to the human reference sequence (NCBI Build 36) and paired using SOLiD System Analysis Pipeline Tool Bioscope, allowing up to 12 color code mismatches per 50 bp tag. For sample CD5 and CD8, two DNA size fractions were merged for library construction which resulted in a reduced sensitivity to identify small deletions.

### DNA-PET Data Curation

The majority of the PET sequences mapped accordingly to the reference genome (concordant PETs or cPETs) with expected mapping orientation (5′ tag to 3′ tag) and expected mapping distance (according to the selected fragment size) The distribution of cPET in the genome was used to retrieve copy number information.^5^ The remaining portion of the PETs mapped discordantly to the reference genome (discordant PET or dPETs), classified as those with incorrect paired-tag orientation and incorrect genomic distances. These dPETs provided information to search for genomic rearrangements; with specific criteria for different types of SVs according to PET mapping orientation and genomic region as described by Yao et al. [19]. The overlapping dPETs representing similar SVs were clustered together as dPET clusters and counted as the cluster size, according to the procedure as described in Hillmer et al. [17] with refined data curation as described in Ng et al. [20].

### Filtering of Normal Structural Variations (SVs)

Comparison of clusters across different genomes was performed as described by Ng et al. [20]. We included DNA-PET data of 23 normal individuals (25 DNA-PET data sets) and the pilot release set of 1000 Genome Project [22] from Mills et al. in the cross-genome comparison, and identified SVs that were present in the normal libraries. In addition, we used the breakpoint locations to compare the identified SVs with published SVs based on paired-end sequencing studies of 18 additional normal individuals [24], [25] and Database of Genomic Variants. The fraction of predicted SV which overlapped with a published SV was calculated by the percentage of overlap relative to the larger event. Thus, we categorized SVs that overlapped by 80% or more with those identified by these studies as normal SVs. Hence, SVs classified as normal have been excluded to identify rearrangements which underlie the diseases.

### CNVs Screening in Public Datasets

We used the CNVs map from published data by Cooper et al. with 15,767 cases of DD and 8,329 adult controls to screen for deletions or duplications in our candidate genes [28]. We also screened from DECIPHER databases, with >17,000 cases carrying CNVs disrupting individual genes. For additional control dataset, we screened for normal CNVs in the first release SVs set of 1000 Genome Project Consortium of 185 individuals [22]. CNVs were counted in both cases and controls spanning candidate genes in these datasets.

### *In silico* Analysis of Regulatory Regions

The hg18 coordinates of genomic regions from each patient’s SVs list were converted to hg19 in LiftOver from UCSC genome browser. SVs coincide within introns or intergenic regions were assessed for the probability score of functional regulatory regions in RegulomeDB [56] and ENCODE data [57], [58] in UCSC genome browser.

### Validations of Expected Breakpoints by PCR

Primers were designed by Primer3 program, and the amplicons spanning the breakpoint were predicted by dPET clusters according to human genome assembly NCBI Build 36. PCR was carried out with JumpStart REDAccuTaq LA polymerase (Sigma Aldrich Inc., St. Louis, MO) in a 50 µl reaction volume and with 500 ng of genomic DNA as a template. The following program was used: 1) Initial denaturation at 96°C for 30 sec, 2) 40 cycles of 15 sec at 94°, 30 sec at 58°C, 10 min at 68°, 3) 68°C for 10 min. PCR products showing single bands were purified by Gel Extraction Kit (Qiagen) and used as templates for sequencing in both directions by Sanger sequencing. The sequences of junction fragments were aligned to the human genome reference sequence using Blat [59].

### Expression Analysis

Total RNA was extracted from patient’s EBV-LCL or fibroblasts using RNAeasy kit (Qiagen). Reverse transcription of 2 µg RNA derived from patients, 2 lymphoblast controls, 2 fibroblast controls and commercially available human tissue panel RNA (Clontech) was performed in 20 µl of SuperSCript III Reverse Transcriptase reaction buffer (Life Technologies) using random hexamer primers. Quantitative real-time PCR (qRT-PCR) reactions were performed in the ABI PRISM 7500 HT system (Life Technologies) with five-fold dilution of cDNA, 200 nM of each primer using the SybrGreen PCR Master Mix (Life Technologies). Data were analyzed using 2^ΔΔCt^ method, and normalized against control sample with human *ACTB*. Each measurement was performed in triplicate. The controls used in this study are derived from EBV-LCL or skin fibroblast cells of 4 normal individuals.

## Supporting Information

Information S1 The file *SupportingInformation_S1.pdf* contains additional information to the manuscript. It consists of 9 pages, 1 Figure and 7 tables.
(DOCX)
