# Supplementary material for: Detection of Chromosomal Breakpoints in Patients with Developmental Delay and Speech Disorders
Source: PLoS One. 2014 Mar 6;9(3):e90852. doi: 10.1371/journal.pone.0090852 (PMC3946304; doi:10.1371/journal.pone.0090852)
Supplement: Information S1 — The file SupportingInformation_S1.pdf contains additional information to the manuscript. It consists of 9 pages, 1 Figure and 7 tables. (DOCX) [file pone.0090852.s001.docx]

**Supporting Information S1.**

**Detection of Chromosomal Breakpoints in Patients with Developmental Delay and Speech Disorders**

Kagistia H. Utami, Axel M. Hillmer, Irene Aksoy, Elaine G. Y. Chew, Audrey S. M. Teo, Zhenshui Zhang, Charlie W.H. Lee, Pauline J. Chen, Chan Chee Seng, Pramila N. Ariyaratne, Sigrid L. Rouam, Lim Seong Soo, Saira Yousoof, Ivan Prokudin, Gregory Peters, Felicity Collins, Meredith Wilson, Alyson Kakakios, Georges Haddad, Arnaud Menuet, Olivier Perche, Stacey Kiat Hong Tay, Ken W.K. Sung, Xiaoan Ruan, Yijun Ruan, Edison T. Liu, Sylvain Briault, Robyn V. Jamieson, Sonia Davila, and Valere Cacheux^#^.

**Figure S1. Workflow of DNA-PET sequencing technique**

**
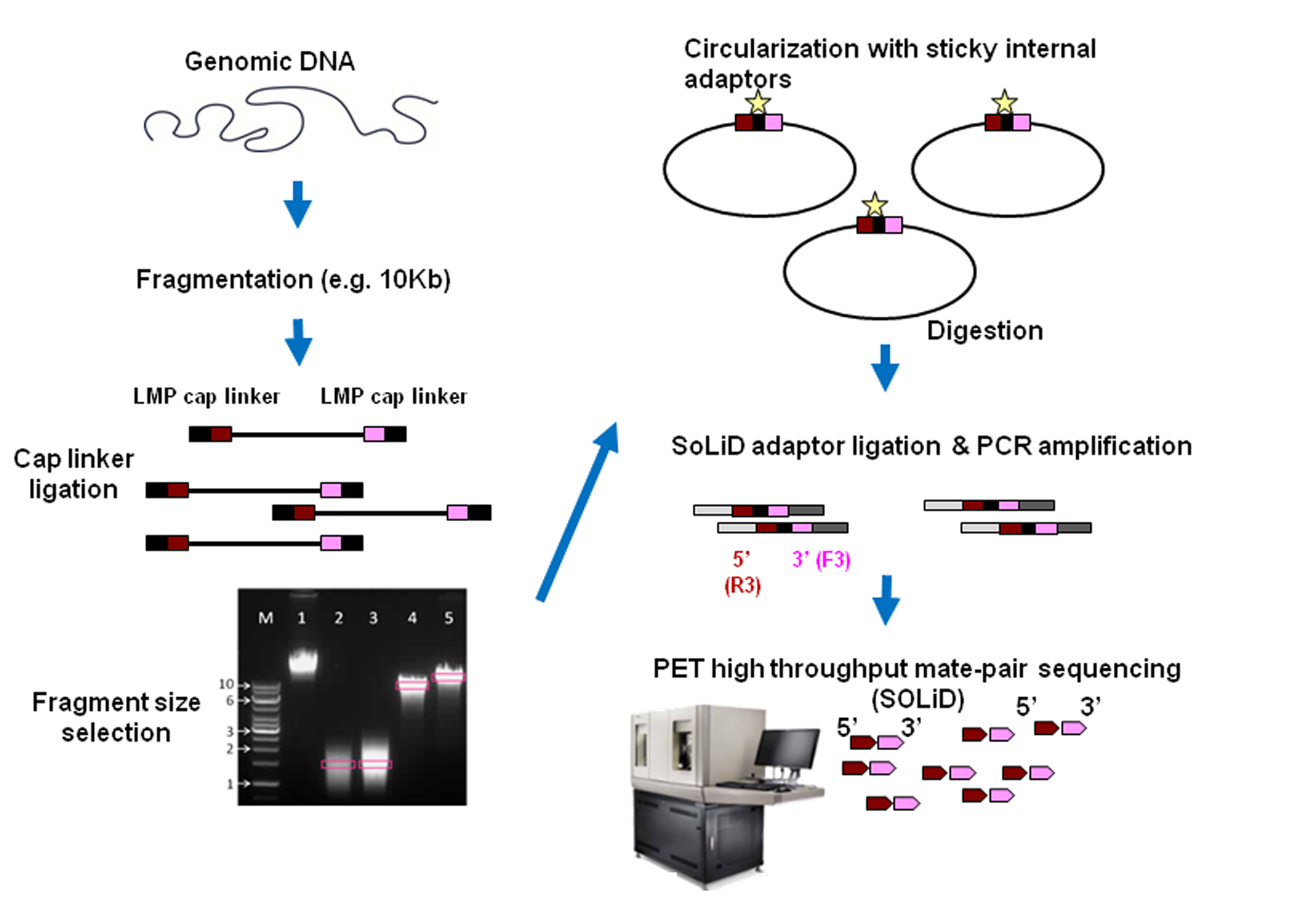
**

Genomic DNA was hydrosheared into a uniform size of approximately 10 kb fragments. Long Mate-Paired (LMP) cap adaptors were ligated to each end of the DNA fragments. The fragments were separated on agarose gel electrophoresis and selected fragment size was used for ligation. This circularization joined the 5’ (dark red) and 3’ (pink) ends of each fragment together by internal biotinylated adaptors. Constructs were digested by nucleases (S1 nuclease and T7 exonucleases) to release the PET constructs and ligated with SOLiD sequencing adaptors. The PET constructs were amplified by PCR and subjected to high-throughput sequencing by SOLiD system.

| Table S1: Statistics of massively parallel PET sequencing on SOLiD platform | | | | | | | | | |
| --- | --- | --- | --- | --- | --- | --- | --- | --- | --- |
| Sample | **Size** | **Tags^a)^** | **Mappable tags^b)^** | **PET^c)^** | **PET (NR)^d)^** | **cPET^e)^**  **span range**  **(bp)** | **cPET (NR)^f)^** | **Coverage^g)^** | **dPET (NR)^h)^** |
| 1 (CD5) | 2 x 50 bp | 359,506,045 | 184,146,758 | 42,680,822 | 18,669,249 | 6,070-15,900 | 15,377,825 | 58.1 | 3,291,424 |
| 2 (CD10) | 2 x 50 bp | 225,470,434 | 142,499,079 | 44,421,203 | 38,775,532 | 7,100-9,230 | 32,994,073 | 93.5 | 5,781,459 |
| 3 (CD8) | 2 x 50 bp | 351,399,088 | 198,212,236 | 57,591,305 | 30,730,151 | 7,470-14,880 | 27,552,231 | 103.0 | 3,177,920 |
| 4 (CD9) | 2 x 50 bp | 229,308,838 | 171,249,332 | 61,252,841 | 54,204,668 | 5,130-10,350 | 40,545,617 | 122.2 | 13,659,051 |

**^a)^**number of sequenced tags; **^b)^**number of mapped tags to the human reference genome (NCBI build 36); **^c)^**number of tags which have been mapped and paired to form paired-end tags (PET); **^d)^**non-redundant PETs; PETs which have the same starting points for both paired tags and thus been excluded based on the assumption that they are derived from the same PCR product (not independent biological information); **^e)^**concordant PETs; **^f)^**non redundant cPETs; **^g)^**physical coverage by cPETs; average number of cPET connections crossing a chromosomal position; **^h)^**non redundant discordant PETs.

| Table S2: List of SVs found in Patient CD5 | | | | | | | | | | |
| --- | --- | --- | --- | --- | --- | --- | --- | --- | --- | --- |
|  |  |  |  |  |  |  |  | **PCR validation** | | |
| SV No | **SV Type^a)^** | **Breakpoint** | **Cluster  Size** | **Span (bp)** | **Gene** | **DNA alteration** | **DGV^b)^** | **Patient CD005** | **Affected son CD020** | **Affected son CD021** |
| 1 | Del | chr1 (+):72,390,624-72,430,488 | 17 | 39,864 | *NEGR1* | Intronic deletion | 28 | + | + | + |
| 2 | Del | chr1 (+):109,337,135-109,357,109 | 15 | 19,974 | *WDR47* | Deleted exon 5-8 | 9 | + | + | + |
| 3 | Del | chr1 (+):114,674,249-114,682,110 | 16 | 7,861 |  |  |  | - | - | - |
| 4 | Del | chr2 (+):31,004,746-31,033,602 | 17 | 28,856 | *GALNT14* |  | 5 | + | + | + |
| 5 | Del | chr4 (+):91,698,589-94,246,096 | 16 | 2,547,507 | *TMSL3* | Full length deletion | 2 | + | - | + |
|  |  |  |  |  | *GRID2* | Deleted exon 1-2 | 46 |  |  |  |
| 6 | Del | chr4 (+):122,847,359-122,891,237 | 16 | 43,878 |  |  |  | + | + | + |
| 7 | Del | chr6 (+):89,226,451-89,232,156 | 7 | 5,705 |  |  |  | + | + | - |
| 8 | Del | chr7 (+):16,201,711-16,211,601 | 25 | 9,890 |  |  |  | + | + | - |
| 9 | Del | chr7 (+):142,534,798-142,6045,66 | 16 | 69,768 | *TAS2R39* | Full length deletion | 3 | + | - | - |
|  |  |  |  |  | *PIP* | Full length deletion | 67 |  |  |  |
| 10 | BT | chr9 (+):79,571,716 | 16 | NA | *GNAQ* | Breakpoint at intron 5 |  | + | + | + |
|  |  | chr17 (+):74,768,401 |  |  | *RBFOX3* |  |  |  |  |  |
| 11 | BT | chr9 (-):79,573,787 | 14 | NA | *GNAQ* | Breakpoint at intron 2 |  | + | + | + |
|  |  | chr17 (-):74,764804 |  |  | *RBFOX3* |  |  |  |  |  |
| 12 | Del | chr11 (+):7,643,628-7,653,635 | 6 | 10,007 | *CYB5R2* | Deleted exon 4-7 | 1 | - | - | - |
| 13 | Del | chr15 (+):59,473,758-59,487,405 | 28 | 13,647 |  |  |  | + | + | + |
| 14 | Del | chr16 (+):4,072,901-4,082,506 | 7 | 9,605 | *ADCY9* | Intronic deletion | 6 | - | - | - |

^a^) Abbreviations are as follows: Del = Deletion; BT= Balanced Translocation

^b^) Number of variant entries present in the DGV for a given gene

| Table S3: List of SVs found in Patient CD10 | | | | | | | |
| --- | --- | --- | --- | --- | --- | --- | --- |
| SV No | **SV Type^a)^** | **Breakpoint** | **Cluster  Size** | **Span (bp)** | **Gene** | **DNA alteration** | **DGV** |
| 1 | Del | chr1 (+):202,173,929-202,188,904 | 27 | 14,975 |  |  |  |
| 2 | Del | chr2 (+):139,455,505-139,459,801 | 29 | 4,296 |  |  |  |
| 3 | Ins | chr2 (+):178,545,739; chr12 (+):54,068,618 | 14 | NA | *PDE11A* | Disruption intron 1 | 18 |
| 4 | Ins | chr2 (-):178,562,405; chr12 (-):54,070,437 | 6 | NA | *PDE11A* | Disruption intron 1 | 18 |
| 5 | Del | chr4 (+):92,499,237-92,504,340 | 29 | 5,103 | *FAM190A* | Intronic deletion | 79 |
| 6 | Del | chr6 (+): 34,511,385- 34,517,410 | 31 | 6,025 |  |  |  |
| 7 | BT | chr6 (+):98,318,059; | 36 | NA |  |  |  |
|  |  | chr8 (-):35,527,808 |  |  | *UNC5D* | Breakpoint at intron 2 |  |
| 8 | BT | chr6 (-):98,318,840; | 34 | NA |  |  |  |
|  |  | chr8 (+):35,528,282 |  |  | *UNC5D* | Breakpoint at intron 2 |  |
| 9 | IB | chr7 (-):217,017; chr15 (-):100,042,752 | 48 | NA | *TARSL2* | Disruption intron 11 |  |
| 10 | Del | chr7 (+):5,846,915-5,855,247 | 13 | 8,332 | *ZNF815* | Deleted exon 4-6 | 6 |
| 11 | Del | chr7 (+):11,886,866-11,889,627 | 26 | 2,761 |  |  |  |
| 12 | Del | chr7 (+):100,369,276-100,374,934 | 22 | 5,658 |  |  |  |
| 13 | TD | chr10 (+): 84,405,124-84,414,680 | 30 | 22,078 | *NRG3* | Intronic duplication | 54 |
| 14 | Del | chr11 (+):62,267,054-62,270,934 | 13 | 3,880 |  |  |  |
| 15 | Del | chr15 (+):48,758,881-48,764,977 | 32 | 6,096 | *TRPM7* | Intronic deletion | 2 |

^a^) Abbreviations are as follows: IB= Isolated breakpoint; Ins=Insertion; TD=Tandem Duplication

| Table S4: List of SVs found in Patient CD8 | | | | | | | |
| --- | --- | --- | --- | --- | --- | --- | --- |
| SV No | **SV Type** | **Breakpoint** | **Cluster  Size** | **Span (bp)** | **Gene** | **DNA alteration** | **DGV^*^** |
| 1 | TD | chr1 (+) :143,753,709-143,758,374 | 25 | 12,876 | *PDE4DIP* | Intronic duplication | 0 |
| 2 | Del | chr4 (+) :32,973,945-32,982,487 | 6 | 8,542 |  |  |  |
| 3 | Del | chr5 (+) :97,026,615-97,046,690 | 22 | 20,075 |  |  |  |
| 4 | Del | chr5 (+) :113,354,357-113,364,703 | 45 | 10,346 |  |  |  |
| 5 | Del | chr7 (+) :12,986,617-12,995,338 | 24 | 8,721 |  |  |  |
| 6 | TD | chr8 (+) :128,450,797-128,413,587 | 46 | 58,701 |  |  |  |
| 7 | Del | chr8 (+) :16,778,010-16,786,728 | 6 | 8,718 |  |  |  |
| 8 | Del | chr9 (+) :90,123,341-90,127,712 | 12 | 4,371 |  |  |  |
| 9 | Del | chr14 (+) :83,112,675-83,120,540 | 56 | 7,865 |  |  |  |
| 10 | TD | chr19 (+) :43,243,485-43,164,809 | 16 | 96,613 | *SIPA1L3* | Intronic duplication | 4 |
| 11 | Del | chr20 (+) :58,544,674-58,558,612 | 40 | 13,938 |  |  |  |
| 12 | TD | chr22 (+) :49,417,248-49,424,209 | 17 | 8,643 |  |  |  |
| 13 | Del | chrX (+) :120,059,370-120,309,394 | 30 | 250,024 |  |  |  |
| 14 | UI | chrX (-):34,649,915; (+)125,672,128 | 49 | 91,022,213 |  |  |  |
| 15 | PI | chrX (+):119,984,844; (+)122,845,538 | 26 | 2,854,430 | *XIAP* | Breakpoint at intron 1 |  |
| 16 | PI | chrX (-):119,997,337; (-)122,856,765 | 29 | 2,860,694 | *XIAP* | Breakpoint at intron 1 |  |
| 17 | TD | chrX (+) : 34,569,874-123,107,520 | 23 | 88,554,667 | *TMEM47* | Part of complex inversion |  |
| 18 | TD | chrX (+) : 123,127,427-125,658,649 | 30 | 2,555,127 | *ODZ1* | Part of complex inversion |  |
|  |  |  |  |  | *SH2D1A* |  |  |
| 19 | TD | chrX (+) : 125,110,988-125,828,479 | 26 | 737,420 | *CXorf64* | Part of complex inversion |  |

^a^) Abbreviation: PI= Paired Inversion; UI=Unpaired Inversion

| Table S5. List of SVs found in chromosome X of Patient CD8 including lower confidence cluster (cluster size ≥ 4) | | | | | | | | | | | |
| --- | --- | --- | --- | --- | --- | --- | --- | --- | --- | --- | --- |
|  | **DNA-PET SVs** | | | **FISH validation** | | | |  | **PCR validation** | | |
| SV^a)^ | **SV Type** | **Cluster Size** | **Span (bp)** | **Breakpoint A** | **BAC Probe**  **for Bp A** | **Breakpoint B** | **BAC Probe for Bp B** | **Gene** | **Patient** | **Affected twin** | **Unaffected mother** |
| 13 | Del | 30 | 250,024 | chrX (+) :120,059,370 | - | chrX (+) :120,309,394 | - |  | + | + | + |
| 14 | UI | 49 | 91,022,213 | chrX (+) : 34,649,915 | - | chrX (-) :125,672,128 | - |  |  |  |  |
| 15 | PI | 26 | 2,854,430 | chrX (+) :119,984,597 | RP1-296G17 | chrX (-) : 122,839,027 | RP1-315G1 | *XIAP* | + | + | + |
| 16 | PI | 29 | 2,860,694 | chrX (-) :119,984,844 | RP1-296G17 | chrX (+) :122,845,538 | RP1-315G1 | *XIAP* | + | + | + |
| 17 | Dup | 23 | 88,554,667 | chrX (+) :123,107,520 | W12-499N23 | chrX (+) : 34,569,874 | RP11-330K13 | *TMEM47* | + | + | + |
| 18 | Dup | 30 | 2,555,127 | chrX (+) :125,658,649 | - | chrX (+) :123,127,427 | W12-499N23 | *ODZ1* |  |  |  |
|  |  |  |  |  |  |  |  | *SH2D1A* |  |  |  |
| 19 | Dup | 26 | 737,420 | chrX (+) :125,828,479 | - | chrX (+) :125,110,988 | - | *CXorf64* |  |  |  |
| 20* | UI | 5 | 9,146 | chrX (-) :34,558,550 | RP11-330K13 | chrX (+) 34,567,696 | - | *TMEM47* | + | + | + |
| 21* | UI | 5 | 88,548,885 | chrX (+) :34,560,395 | RP11-330K13 | chrX (-) :123,109,280 | W12-499N23 | *TMEM47* | + | + | + |
| 22* | TD | 4 | 28,847 | chrX (+) :61,754,201 | RP11-762M23 | chrX (+) : 61,771,832 | RP11-762M23 |  | - | - | - |

^a^) SV numbers correspond to the SVs listed in table S4

^*^) Lower confidence DNA-PET clusters (CS ≥ 4)

| Table S6: List of SVs found in Patient CD9 | | | | | | | |
| --- | --- | --- | --- | --- | --- | --- | --- |
| SV No | **SV Type** | **Breakpoint** | **Cluster  Size** | **Span (bp)** | **Gene** | **DNA alteration** | **DGV** |
| 1 | Del | chr1 (+):33189,324-33,193,992 | 43 | 4,668 | *RNF19B* | Intronic deletion | 0 |
| 2 | PI | chr5 (+):111,962,591; (-)165,575,819 | 59 | 53,613,228 |  |  |  |
| 3 | PI | chr5 (-):111,963,149; (+)165,576,628 | 63 | 53,613,479 |  |  |  |
| 4 | TD | chr5 (+):641,668-738,952 | 17 | 97,284 | *TPPP* | Duplicated exon 1-3 | 47 |
|  |  |  |  |  | *CEP72* | Full length duplication | 42 |
| 5 | TD | chr9 (+):78,960,669-78,966,871 | 7 | 6,202 |  |  |  |
| 6 | Del | chr9 (+):106,837,792-106,843,557 | 44 | 5,765 |  |  |  |
| 7 | TD | Chr17(+):2,734,890-2,747,456 | 18 | 12,556 | *RAP1GAP2* | Intronic duplication | 17 |

| Table S7. Comparison of copy number estimates by cPETs and aCGH | |
| --- | --- |
| Sample | **Overlap (%)** |
| CD5 | 95.08 |
| CD10 | 91.93 |
| CD8 | 92.76 |
| CD9 | 92.97 |
